# Simulation to determine the approach of transcatheter aortic valve implantation in patients undergoing hemodialysis

**DOI:** 10.1007/s00595-023-02743-4

**Published:** 2023-09-05

**Authors:** Yusuke Takei, Shunsuke Saito, Ikuko Shibasaki, Toshiyuki Kuwata, Yasuyuki Yamada, Hirotsugu Fukuda

**Affiliations:** 1 Department of Cardiac and Vascular Surgery, Dokkyo Medical University Graduate School of Medicine, 880 Kitakobayashi, Mibu-Machi, Shimotuga-gun, Tochigi, 321-0293 Japan; 2 Division of Cardiovascular Surgery, Maebashi Red Cross Hospital, Maebashi, Gunma Japan; 3 Division of Cardiovascular Surgery, Gunma Prefectural Cardiovascular Center, Gunma, Japan

**Keywords:** Transcatheter aortic valve implantation, Hemodialysis, Vascular complication, Approach simulation

## Abstract

**Purpose:**

The present study investigated potential access vessels in patients receiving hemodialysis who underwent surgical aortic valve replacement and determined which approaches were most suitable for performing transcatheter aortic valve implantation.

**Methods:**

Consecutive patients undergoing hemodialysis with aortic valve stenosis who underwent surgical aortic valve replacement were included. Preoperative computed tomography data were analyzed to assess the vessel diameter and calcification. Simulations were conducted to determine the feasibility of inserting the 14-F eSheath of Sapien 3 via transfemoral, trans-cervical, trans-subclavian, and direct aorta approaches.

**Results:**

A total of 72 patients were included in this study. The access route was characterized by severe calcification of the common iliac artery. The transfemoral approach was feasible in 77.8% of the cases, but the rate decreased to 33% when the calculations were based on the maximum sheath extension diameter. The trans-cervical, trans-subclavian, and direct aortic approaches were suitable for many patients. Lower extremity artery disease was identified as a risk factor for the unsuitability of the transfemoral approach.

**Conclusions:**

Common iliac artery calcification in patients undergoing hemodialysis restricts the use of the transfemoral approach. Therefore, some patients require alternative approaches.

**Supplementary Information:**

The online version contains supplementary material available at 10.1007/s00595-023-02743-4.

## Introduction

Recently, transcatheter aortic valve implantation (TAVI) has become a widely used, well-established procedure for treating patients with aortic valve stenosis (AS) who are at risk for needing surgical aortic valve replacement (SAVR) [1, 2]. This procedure has good outcomes, as documented in the United States nationwide registry [3]. Currently, TAVI is indicated in patients at low risk for surgery as well as in high-risk patients, such as those with end-stage renal dysfunction (ESRD) undergoing hemodialysis (HD). These patients typically have multiple comorbidities, including diabetes, hypertension, heart disease, and lower extremity artery disease (LEAD), which can increase the risk of complications and death intra- and post-operatively [4]. For patients receiving HD, arterial calcification plays a critical role in determining the TAVI site, and a larger proportion of these patients are expected to require alternative approaches than patients not undergoing HD.

At present, the transfemoral (TF) approach is the one most commonly used in TAVI, being characterized by thinner delivery catheters characteristic of third-generation devices than previous generation devices, allowing 95% of procedures to be performed via this approach [3]. While alternative approaches are often avoided, they can offer both advantages and disadvantages in terms of the 30-day mortality, stroke, and hemorrhaging compared with the TF approach [5]. The intricate nature of these procedures may lead to adverse outcomes in patients undergoing HD compared with those not undergoing HD.

Although data on TAVI outcomes in patients receiving HD remain limited, a recent systematic review revealed that patients with ESRD on HD who underwent TAVI are less likely to be treated via the TF approach than patients without HD who underwent TAVI. Patients are also likely to experience higher short- and long-term mortality rates and a higher risk of post-TAVI adverse events, including major bleeding, pacemaker implantation, and device failure, than those not on HD [6]. Over half of patients receiving HD who underwent TAVI with a second-generation device had an alternative approach selected [7, 8].

The features of arterial calcium accumulation in older, diminutive Asian patients with AS undergoing HD and the prevalence of various TAVI approaches in this population are yet to be elucidated. TAVI is currently performed in a limited number of centers in Japan for select patients receiving HD; therefore, it is important to understand this population as TAVI becomes more common in patients undergoing HD. This information may help determine whether or not this high-risk group should undergo TAVI and, if so, by which approach.

The present study (i) investigated the potential access routes in patients on HD who underwent SAVR and (ii) simulated the approaches that would have been chosen if these patients had received TAVI.

## Methods

### Ethical statement

This cross-sectional, single-center study was approved by the Institutional Review Board of Dokkyo Medical University (protocol number R-62-10J) on September 16, 2022. The study was performed in accordance with the ethical standards laid down in the 1964 Declaration of Helsinki and its later amendments. The requirement for individual consent was waived. However, the opt-out declaration form was in the public domain. Patients who wished to withdraw from the study were also excluded.

### Patient selection and study design

Consecutive AS patients with ESRD on HD undergoing SAVR, including concomitant procedures, at Dokkyo Medical University Hospital between June 2008 and August 2021, were eligible for this study. Patients who underwent concomitant mitral valve replacement for mitral stenosis were excluded because transmitral valve replacement was not approved. Patients with incomplete medical records or computed tomography (CT) data were excluded from the study.

Patients’ preoperative characteristics and information on the implanted valves were collected by reviewing their medical records. Preoperative contrast or plain CT data, if contrast CT was not performed, were analyzed for each patient according to the criteria described in the “CT Measurements” section below. The most suitable approach was simulated based on the feasibility of inserting the eSheath of a Sapien 3 (Edwards Lifesciences, Irvine, CA, USA), which was approved for use in TAVI for patients undergoing HD in Japan in 2021.

### CT measurements: vessel diameter and calcification assessments

Measurements were obtained from CT scans of the ascending aorta (Asc. Ao), bilateral common carotid artery (CCA), left subclavian artery (LSA), abdominal aorta (Abd. Ao), bilateral common iliac artery (CIA), external iliac artery (EIA), and common femoral artery (CFA). The following parameters were assessed: (i) minimum short-axis diameter of the vessels, excluding the Asc. Ao; (ii) degree of calcification at the presumed site of sheath insertion for Asc. Ao and at the site of minimal vascular diameter for the other vessels; and (iii) range of calcification in the measured vessels. Both the degree (D) and range (R) of calcification were categorized into 6 scales, as follows: D0 = 0, 0 < D1 ≤ 25%, 25 < D2 ≤ 50%, 50 < D3 ≤ 75%, 75 < D4 ≤ 99%, and D5 = 100%; R0 = 0, 0 < R1 ≤ 25%, 25 < R2 ≤ 50%, 50 < R3 ≤ 75%, 75 < R4 ≤ 99%, and R5 = 100%.

All data acquired from CT scans were analyzed using the OsiriX MD software program, version 12.0.3 (Pixmeo Sàrl, Bernex, Switzerland).

### Approach simulation (Fig. 1)

**Fig. 1:**
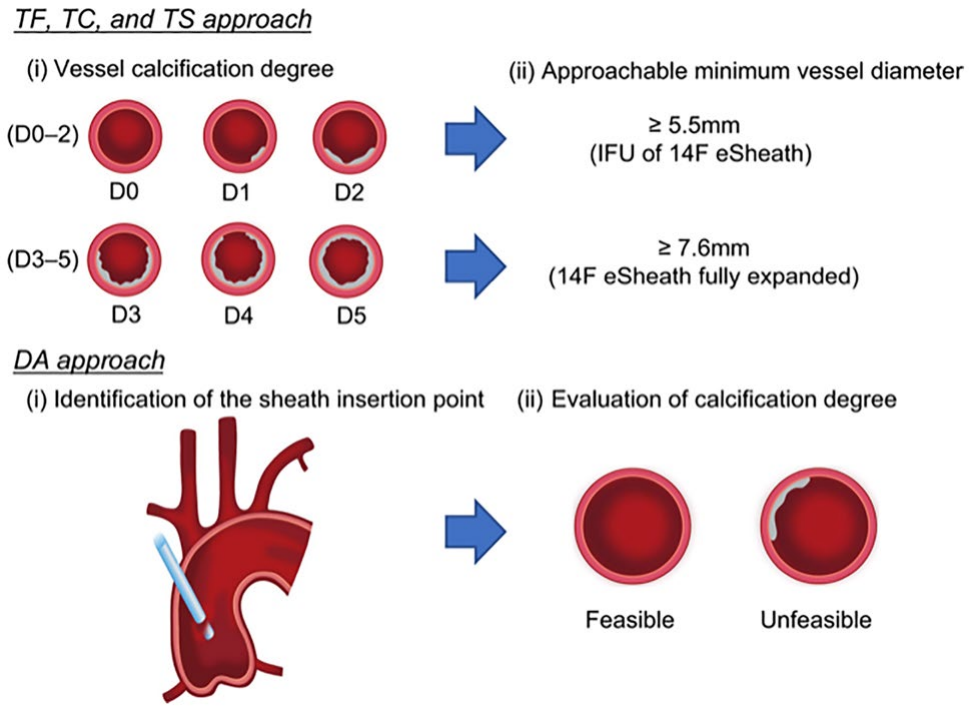
Approach simulation flow. The minimum vessel diameter requirement was determined according to the degree of calcification, and the feasibility of the TF, TC, and TS approaches was determined by whether or not the 14-F eSheath could pass through. The DA approach was based on the presence or absence of calcification at the sheath insertion site. *TF* transfemoral, *TC* transcervical, *TS* transsubclavian, *IFU* instruction for use, *DA* direct aorta

The cardiac structure (valve orifice area, sinus of Valsalva diameter, coronary artery location, valve calcification adherence, etc.), vessel tortuosity, age, and frailty are all factors typically assessed by the Heart Team to determine whether or not TAVI can be performed. However, these factors were not considered in the present study. The simulation proceeded based on the assumption that TAVI was possible in all recruited patients.

An eSheath is a unique sheath that expands as the delivery catheter passes through it. Reportedly, the 14-F eSheath for 20-, 23-, and 26-mm Sapien 3 can expand from 6 to 7.6 mm, while the 16-F eSheath for 29-mm Sapien 3 can expand from 6.7 to 8.2 mm [9]. The selection of the appropriate size of Sapien 3 depended on the size of the SAVR valve to be implanted. According to the size chart for the 29-mm Sapien 3, the target annulus diameter was 26.2–29.5 mm. However, a 27-mm bioprosthetic valve in the intra-annular position in patients with AS undergoing HD was not implanted; thus, we excluded the 16-F eSheath from this study. The simulation was based solely on the vessel diameter. Although the degree of calcification was considered, this was not the case. We established the criteria for the TAVI approach depending on the degree of calcification. We determined whether or not the 14-F eSheath could pass through in each case using the TF, trans-cervical (TC), and trans-subclavian (TS) approaches, or via the direct aorta (DA) approach if insertion was possible. Regarding the specifics of these criteria, if all approaches were deemed unfeasible except for the TC approach, which is not in the instructions for use (IFU), the transapical (TA) approach was selected.

### The evaluation of the TF, TC, and TS approaches

In cases of mild calcification (≤ D2), the IFU of the 14-F eSheath required an access route diameter > 5.5 mm. In cases of moderate or severe calcification (≥ D3), patients with an access route vessel diameter > 7.6 mm, which is the maximum dilation of the 14-F eSheath, were considered suitable. When assessing the feasibility of the TF, TC, and TS approaches, the simulation also examined whether or not a fully dilated 7.6 mm 14-F eSheath could pass through and whether or not a puncture method was viable for the TF approach. The absence of calcification on the anterior and lateral sides of the CFA puncture site indicated that a patient could undergo the TF approach using this puncture method.

### DA approach simulation

After confirming the absence of calcification or atheroma in the Asc. Ao, especially in the aortic outer curvature 5.5–6 cm from the aortic valve annulus, the DA approach was possible.

Online Resource 1 shows an example of a CT measurement in a patient, where the TF, TC, TS, and DA were all determined to be feasible.

### Data collection and statistical analyses

Perioperative, demographic, and operative data were also recorded. There were no missing data for any variable. Categorical variables are represented as counts and percentages, whereas continuous values are expressed as the mean (standard deviation) or median (interquartile range). A sub-analysis was conducted to compare patients deemed suitable for the TF approach with those considered unsuitable for the TF approach based on the simulation. After assessing normality using the Kolmogorov–Smirnov test, the means and medians between the groups were compared using an unpaired Student’s *t* test for normally distributed variables and the Mann–Whitney U-test for non-normally distributed variables. Categorical variables were compared using chi-square or Fisher’s exact tests. To identify risk factors, an age-, sex-, and body surface area-adjusted logistic regression analysis was performed with unsuitability for the TF approach as the dependent variable.

All analyses were performed using the SPSS software program, version 27 (IBM Corp., Armonk, NY, USA); two-tailed *P* values of 0.05 were considered significant.

## Results

### Patient demographics

**Fig. 2:**
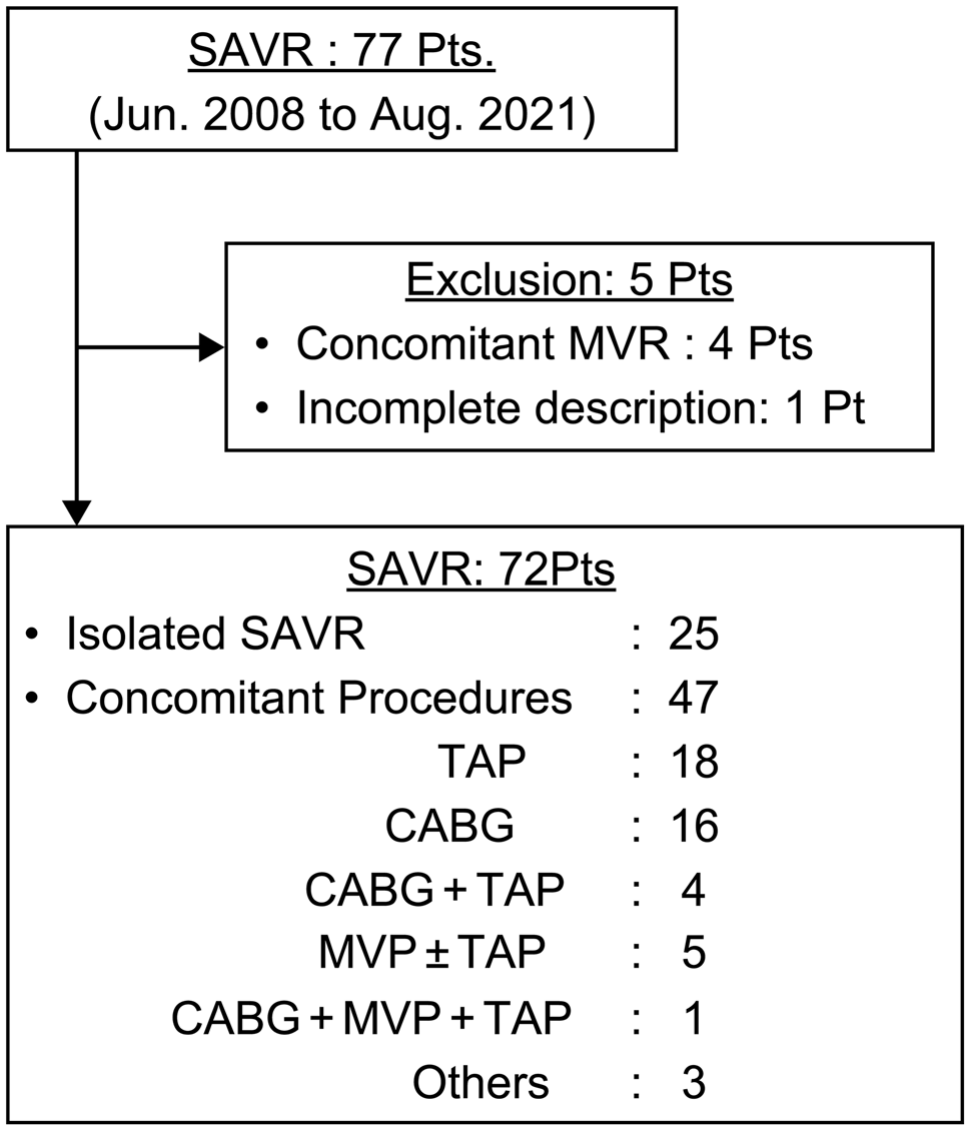
Patients included in the study and concomitant procedures performed. *SAVR* surgical aortic valve replacement, *MVR* mitral valve replacement, *TAP* tricuspid valve annuloplasty, *CABG* coronary artery bypass grafting, *MVP* mitral valve plasty

Between June 2008 and August 2021, 77 patients with AS undergoing HD underwent SAVR at our institution. Based on our exclusion criteria, five patients were excluded from the analysis: four who underwent concomitant mitral valve replacement for mitral valve stenosis and one lacking CT data. Ultimately, 72 patients were included in this study, 47 of whom underwent concomitant procedures, most commonly tricuspid valve annuloplasty or coronary artery bypass grafting (Fig. 2).

Table 1 summarizes the patients’ demographics, revealing a mean age of 72 ± 7.3 years old. Notably, HD was due to diabetic nephropathy in 43.1% of patients; the median time from HD initiation to SAVR was 9 years, and 18.1% of patients had LEAD. Only three patients were implanted with a mechanical valve because of their age; the most commonly implanted valve size was 21 mm (40.3%) via supra-annular implantation.

**Table 1:** Patient demographics and type and size of SAVR valve

	N = 72
Age, years (SD)	72.2 (7.3)
Male, n (%)	43 (59.7)
BSA, m^2^ (SD)	1.4 (0.5)
BMI, kg/m^2^ (SD)	20.5 (3.4)
Diabetic nephropathy, n (%)	31 (43.1)
HD duration, years [IQR]	9 [4–16]
Hypertension, n (%)	51 (70.8)
Current smoker, n (%)	7 (9.7)
Cardiac artery disease, n (%)	28 (38.9)
Cerebral vascular disease, n (%)	10 (13.9)
Lower extremity artery disease, n (%)	13 (18.1)
Implanted surgical valve	
Mechanical valve, n (%)	3 (4.2)
Tissue valve, n (%)	69 (95.8)
Size of implanted surgical valve	
19 mm, n (%)	23 (31.9)
21 mm, n (%)	29 (40.3)
23 mm, n (%)	16 (22.2)
25 mm, n (%)	4 (5.6)

*SD* standard deviation, *BSA* body surface area, *BMI* body mass index, *HD* hemodialysis, *IQR* interquartile range, *SAVR* surgical aortic valve replacement

### Diameter and calcification assessments

**Fig. 3:**
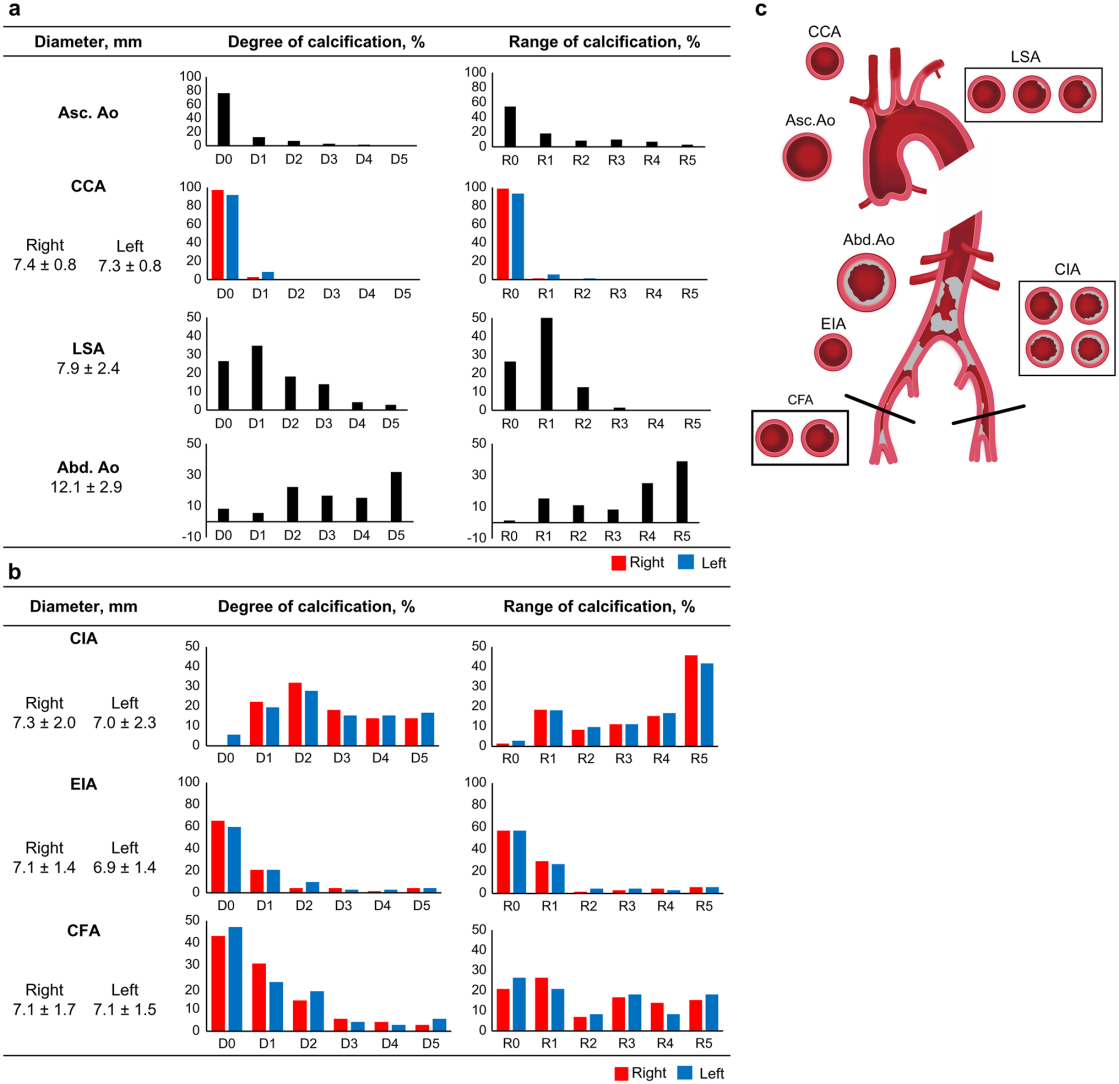
Mean diameter of target vessels the degree and extent of calcification, and aortic calcification image. **a** Asc. Ao, CCA, LSA, and Abd. Ao aorta. **b** CIA, EIA, and CFA. D and R of calcification were categorized into 6 scales each, as follows: D = 0, 0 < D1 ≤ 25%, 25 < D2 ≤ 50%, 50 < D3 ≤ 75%, 75 < D4 ≤ 99%, and D5 = 100%; R = 0, 0 < R1 ≤ 25%, 25 < R2 ≤ 50%, 50 < R3 ≤ 75%, 75 < R4 ≤ 99%, and R5 = 100%. **c** Aortic calcification image created from data. *Asc. Ao* ascending aorta, *CCA* common carotid artery, *LSA* left subclavian artery, *Abd. Ao* abdominal aorta, *CIA* common iliac artery, *EIA* external iliac artery, *CFA* common femoral artery, *D* degree, *R* range

Figure 3 presents the mean minimum diameter of each vessel, the D and R of calcification, and the aortic calcification image (see Online Resource 2 for details of the CT procedure and calcification data). In approximately 88% of the patients, the Asc. Ao at the sheath insertion site exhibited either no (D0) or minor (D1) calcification. The bilateral CCAs were virtually free from calcification. Although the mean diameter of the LSA was sufficiently large for eSheath passage, approximately 20% of LSAs had calcification more severe than D3, particularly at the LSA orifice. The Abd. Ao showed nearly circumferential calcification (D4 and D5) in almost half of the patients, with the entire Abd. Ao involved in approximately 60% of patients (R4 and R5). Almost half of the bilateral CIAs had calcification of more than half the circumference (≥ D3), and approximately 60% of patients exhibited calcification extending over the entire CIA area (≥ R4). The mean minimal diameter, defined by this calcification, was 7.3 mm on the right and 7.0 mm on the left. Conversely, the EIAs tended to show less calcification than the CIAs in both the D and R. In the CFAs, approximately 70% of patients had minor calcification (≤ D1), and approximately 50% exhibited calcification of over 50% of the CFA (≥ R3), with calcification localized near the femoral bifurcation and less calcification closer to the inguinal ligament.

### Approach simulation

The simulation results are listed in Table 2. The TF approach was suitable for 77.8% of patients, with the puncture method feasible in 69.4% of these TF-suitable patients. TS and DA procedures were feasible in 77.8% and 79.2% of the patients, respectively. Although the TC approach is outside the IFU, it was judged to be feasible in 95.8% of patients. Among approximately 20% of patients for whom the TF approach was not recommended, only 1 patient was deemed suitable for the TA approach. When the simulation was conducted using a required vessel diameter of 7.6 mm as the maximum expanded diameter, the TF, TC, and TS approaches were practicable in 33.3%, 55.6%, and 56.9% of the patients, respectively (Table 3). In a subanalysis of patients suitable and unsuitable for the TF approach, LEAD was more common in the TF-unsuitable group than the TF-suitable group (Online Resource 3). In addition, in the logistic regression analysis, LEAD emerged as a risk factor for TF unsuitability (Table 4).

**Table 2:** TAVI approach simulation

	N = 72
Suitable for TF approach, n (%)	56 (77.8)
Bilateral approach, n (%)	42/56 (75.0)
Puncture approach, n (%)	39/56 (69.4)
Available for TC approach, n (%)	69 (95.8)
Available for TS approach, n (%)	56 (77.8)
Available for DA approach, n (%)	57 (79.2)
Desirable for alternative approach, n (%)	16 (22.2)
Preferable to avoid TS approach, n (%)	5 (31.3)
Available for only TA approach^a^, n (%)	1 (6.3)

*TAVI* transcatheter aortic valve implantation, *TF* transfemoral, *TC* transcervical, *TS* trans subclavian, *DA* direct aorta

^a^If the TC approach is excluded

**Table 3:** TAVI approach simulation with a fully expanded eSheath

	N = 72
Suitable for TF approach, n (%)	24 (33.3)
Bilateral approach, n (%)	15/24 (62.5)
Available for TC approach, n (%)	40 (55.6)
Available for TS approach, n (%)	41 (56.9)

*TAVI* transcatheter aortic valve implantation, *TF* transfemoral, *TC* transcervical, *TS* trans subclavian

**Table 4:** Results of a logistic regression analysis on TF approach unsuitability

Variable	OR	95% CI	*P* value
LEAD	5.4	1.0–27.7	.045
Diabetic nephropathy	1.8	0.4–7.6	.43
HD duration	1.0	0.9–1.1	.71
Serum calcium	0.9	0.5–2.3	.90
Serum phosphorus	0.4	0.8–1.8	.36

Adjusted for age, sex, and body surface area

*TF* transfemoral, *LEAD* lower extremity arterial disease, *HD* hemodialysis, *OR* odds ratio, *CI* confidence interval

## Discussion

The main findings of the present study are as follows: (1) Patients on HD exhibited low calcification of the Asc. Ao and CCA and a sufficient LSA diameter despite occasional calcification at its origin. The CIAs had extensive and severe calcification, whereas the EIAs had minimal calcification. Most CFAs present with mild calcification, particularly near the femoral artery bifurcation. (2) According to the access simulation, the TF approach was feasible in 77.8% of patients. However, when the maximum sheath extension diameter was considered, it decreased to approximately 30% of patients. In contrast, alternative approaches, such as via the TC, TS, and DA, were deemed feasible for a high proportion of patients based on the diameter and calcification of the access vessel. (3) As expected, LEAD was a risk factor for TF approach unsuitability, and no HD-specific risk factors were identified in this study.

An annual report by the United States Renal Data System [10] in 2020 revealed that Taiwan, the Republic of Korea, and Japan had the highest incidences of treated ESRD. In addition, 5 of the 8 countries with a prevalence exceeding 2000 per 1 million people are in Asia. In Japan, the average age at HD initiation is approximately 65 years old, and approximately 8% of patients on HD have been undergoing HD for ≥ 20 years due to effective clinical and social management [11]. In contrast, an estimated 2–4% of patients with AS also undergo HD [12], and data from the United States showed that 4% of TAVI patients were on HD [13]. Given this context, the demand for TAVI treatment for patients receiving HD is expected to increase, raising questions about whether or not current devices can be used via the same TF approach for these patients as for those not receiving HD, especially among Asian patients with their relatively small body sizes.

Vascular calcification forms in the intima and media of arteries, the latter being characteristic of HD [14], particularly due to chronic disease-mineral and bone disorders [15]. A previous study reported that patients on HD who have undergone TAVI exhibit vascular calcification more frequently than those not on HD, with a severe calcium burden observed in the descending aorta, infrarenal aorta, and iliofemoral arteries [16]. In the present study, many patients had extensive and circumferential calcifications of the Abd. Ao and CIA. It is reasonable to assert that the feasibility of the TF approach depends on the degree of calcification of CIA. In our simulation, the TF approach was considered feasible in 77.8% of cases. Conversely, in a Japanese clinical trial [17] using the third-generation TAVI device Sapien 3, the TF approach was used in 89.3% of cases, with the TA approach applied in the remaining cases, although the number of cases was small. However, there is the possibility of selection bias in determining the feasibility of the TF approach. If TAVI is performed more widely in patients on HD in the future, it is expected that TAVI will be performed via the TF approach in a smaller proportion of patients on HD than in those not on HD.

Based on our study, we believe that alternative approaches such as TC, TS, and DA are possible. However, how the TS approach affects access to blood vessels that are commonly created on the left-hand side is unclear. Furthermore, the TC approach was outside the IFU of the eSheath. Nevertheless, the reported positive results of the TS [18] and TC [19] approaches and the vascular characteristics of patients on HD suggest that these may be feasible as relatively minimally invasive approaches.

Vascular complications (VCs), classified as major or minor according to the Valve Academic Research Consortium-3 criteria [20], contribute to poor operative outcomes [21]. With advances in TAVI device delivery systems, the frequency of VCs related to major access sites has decreased to 1.3% [3]. However, a recent study reported that, owing to vascular calcifications, TAVI in patients with ESRD resulted in major access site VCs (6.0%) more frequently than in patients without ESRD [16]. Previous reports have also identified moderate-severe iliofemoral calcifications, tortuosity, the sheath outer-to-iliofemoral artery ratio (SIFAR) [22], and the sheath outer-to-femoral artery ratio (SFAR) as predictors of VCs [23]. The suggested cut-off points for major VCs were 0.95 for the SIFAR and 1.05 for the SFAR. Because these studies used older-generation sheaths rather than the currently used expandable sheaths, the application of these ratios in our model is not straightforward. It should be emphasized that, in the present study, 25% of patients had a SIFAR > 0.95, including the potential for VCs, even in the group suitable for TF, and only approximately 33% of the cases in which the TF approach was considered safe to pass through when the sheath was fully expanded. Vascular calcification, phosphate retention, excess calcium, a history of dialysis, active high-dose vitamin D therapy, and deficiency of calcification inhibitors may all be risk factors [14]. However, the subanalysis of patients divided by TF suitability did not identify these factors of arterial calcification in patients on HD.

Several limitations associated with the present study warrant mention. First, the analysis was conducted at a single center with a small sample size, and the indications for TAVI, such as cardiac structure, age, and comorbidities were not examined; therefore, patients for whom TAVI was not feasible in practice may have been included. Second, we focused solely on the vessel diameter in the simulation. While we considered the degree of calcification, we did not consider the extent of calcification or vessel tortuosity because we did not know to what extent the range of calcification or vessel tortuosity would affect the passage of the sheath. In addition, none of the patients in our study had severe tortuosity. Finally, half of the patients were assessed using non-contrast CT, which was the standard for evaluating the aorta and peripheral arteries prior to the approval of TAVI for patients on HD. This may have led to an overestimation of vascular diameters, making it impossible to assess intravascular atheroma. As a result, we may have overestimated the number of patients who were suitable for TF. Notably, most patients who were unsuitable for TF had calcific stenosis of the CIAs. We measured their diameters using the inside calcification as a reference, which likely minimized the discrepancy in measurement error compared with contrast CT.

## Conclusion

Patients undergoing HD exhibit substantial calcification in the CIAs, which affects the feasibility of the TF approach. TAVI for patients on HD, even with the current expandable devices, will require a significant number of alternative approaches and increasingly require the participation of surgeons.

### Supplementary Information

Below is the link to the electronic supplementary material.

Supplementary file1 Online Resource 1. Example CT measurements and simulation. The degree and range of calcification and smallest vessel diameter were measured at each site. In this case, all approaches were considered feasible. CT: computed tomography, Asc: Asc. Ao: ascending aorta, LSA: left subclavian artery, Abd. Ao, abdominal aorta; CCA, common carotid artery; CIA, common iliac artery; EIA, external iliac artery; CFA, common femoral artery; TF, transfemoral; TS, trans-subclavian; TC, transcervical; DA, direct aorta (PDF 1629 KB)

Supplementary file2 (PDF 217 KB)

Supplementary file3 (PDF 425 KB)

## Data Availability

Data supporting the findings of this study are available from the corresponding author [Y. T.] upon reasonable request.
