# Supplementary material for: Simulation to determine the approach of transcatheter aortic valve implantation in patients undergoing hemodialysis
Source: Surg Today. 2023 Sep 5;54(5):428–35. doi: 10.1007/s00595-023-02743-4 (PMC11026236; doi:10.1007/s00595-023-02743-4)
Supplement: Supplementary file 1 — Supplementary file1 Online Resource 1. Example CT measurements and simulation. The degree and range of calcification and smallest vessel diameter were measured at each site. In this case, all approaches were considered feasible. CT: computed tomography, Asc: Asc. Ao: ascending aorta, LSA: left subclavian artery, Abd. Ao, abdominal aorta; CCA, common carotid artery; CIA, common iliac artery; EIA, external iliac artery; CFA, common femoral artery; TF, transfemoral; TS, trans-subclavian; TC, transcervical; DA, direct aorta (PDF 1629 KB) [file 595_2023_2743_MOESM1_ESM.pdf]

ESM_1

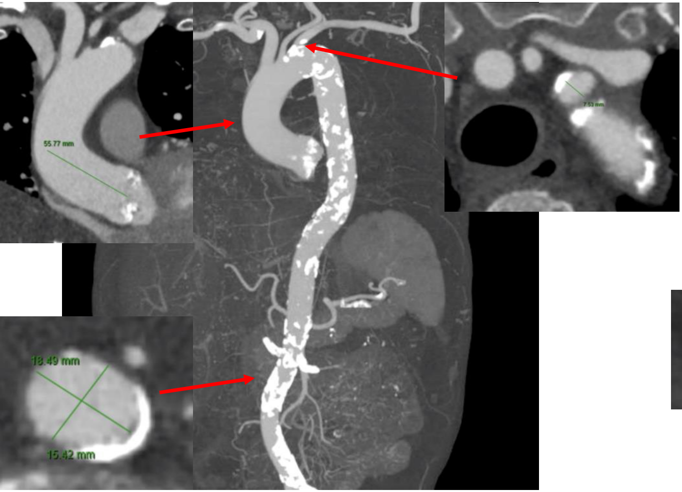

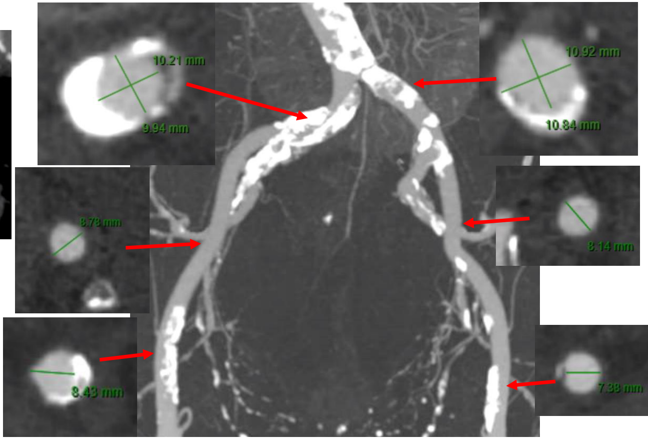

1. CT measurement

Location	Diameter, mm		Calcification (D)		Calcification (R)	
Asc.Ao	-		D0		R0	
LSA	7.6		D1		R1	
Abd.Ao	15.4		D2		R4	
CCA	7.8	7.8	D0	D0	R0	R0
CIA	9.9	10.8	<u>D4</u>	D2	R4	R3
EIA	8.7	8.1	D0	D0	R0	R1
CFA	8.4	7.4	D2	D0	R4	R3

2. Access simulation

- Suitable for bilateral TF approach
- Available for TS, TC, DA approach
