# Supplementary material for: Simulation to determine the approach of transcatheter aortic valve implantation in patients undergoing hemodialysis
Source: Surg Today. 2023 Sep 5;54(5):428–35. doi: 10.1007/s00595-023-02743-4 (PMC11026236; doi:10.1007/s00595-023-02743-4)
Supplement: Supplementary file 2 — Supplementary file2 (PDF 217 KB) [file 595_2023_2743_MOESM2_ESM.pdf]

Online Resource 2

Details of the CT scanning procedure

	n = 72
Contrast CT, n (%)	39 (54.2)
Slice width, n (%)	
1 mm	1 (1.4)
3 mm	2 (2.8)
5 mm	24 (33.3)
8 mm	45 (62.5)

Details of the degree and range of calcification

Vessel	Degree of calcification, n (%)						Range of calcification, n (%)					
	D0	D1	D2	D3	D4	D5	R0	R1	R2	R3	R4	R5
Asc. Ao	55 (76.4)	9 (12.5)	5 (6.9)	2 (2.8)	1 (1.4)	0 (0)	39 (54.2)	13 (18.1)	6 (8.3)	7 (9.7)	5 (6.9)	2 (2.8)
CCA (Right)	70 (97.2)	2 (2.8)	0 (0)	0 (0)	0 (0)	0 (0)	71 (98.6)	1 (1.4)	0 (0)	0 (0)	0 (0)	0 (0)
CCA (Left)	66 (91.7)	6 (8.3)	0 (0)	0 (0)	0 (0)	0 (0)	67 (93.1)	4 (5.6)	1 (1.4)	0 (0)	0 (0)	0 (0)
LSA	19 (26.4)	25 (34.7)	13 (18.1)	10 (13.9)	3 (4.2)	2 (2.8)	19 (26.4)	43 (59.7)	9 (12.5)	1 (1.4)	0 (0)	0 (0)
Abd. Ao	6 (8.3)	4 (5.6)	16 (22.2)	12 (16.7)	11 (15.3)	23 (31.9)	1 (1.4)	11 (15.3)	8 (11.1)	6 (8.3)	18 (25.0)	28 (38.9)
CIA (Right)	0 (0)	16 (22.2)	23 (31.9)	13 (18.1)	10 (13.9)	10 (13.9)	1 (1.4)	13 (18.4)	6 (8.3)	8 (11.1)	11 (15.3)	33 (45.8)

CIA (Left)	4 (5.6)	14 (19.4)	20 (27.8)	11 (15.3)	11 (15.3)	12 (16.7)	2 (2.8)	13 (18.1)	7 (9.7)	8 (11.1)	12 (16.7)	30 (41.7)
EIA (Right)	47 (65.3)	15 (20.8)	3 (4.2)	3 (4.2)	1 (1.4)	3 (4.2)	41 (56.9)	21 (29.2)	1 (1.4)	2 (2.8)	3 (4.2)	4 (5.6)
EIA (Left)	43 (59.7)	15 (20.8)	7 (9.7)	2 (2.8)	2 (2.8)	3 (4.2)	41 (56.9)	19 (26.4)	3 (4.2)	3 (4.2)	2 (2.8)	4 (5.6)
CFA (Right)	31 (43.1)	22 (30.6)	10 (13.9)	4 (5.6)	3 (4.2)	2 (2.8)	15 (20.8)	19 (26.4)	5 (6.9)	12 (16.7)	10 (13.9)	11 (15.3)
CFA (Left)	34 (47.2)	16 (22.2)	13 (18.1)	3 (4.2)	2 (2.8)	4 (5.6)	19 (26.4)	15 (20.8)	6 (8.3)	13 (18.1)	6 (8.3)	13 (18.1)

Degree and range of calcification were categorized into six scales each: D = 0, 0 < D1 ≤ 25%, 25 < D2 ≤ 50%, 50 < D3 ≤ 75%, 75 < D4 ≤ 99%, and D5 = 100%; R = 0, 0 < R1 ≤ 25%, 25 < R2 ≤ 50%, 50 < R3 ≤ 75%, 75 < R4 ≤ 99%, and R5 = 100%. Asc. Ao, ascending aorta; CCA, common carotid artery; LSA, left subclavian artery; Abd.Ao, abdominal aorta; CIA, common iliac artery; EIA, external iliac artery; CFA, common femoral artery; D, degree; R, range
