# Supplementary material for: Simulation to determine the approach of transcatheter aortic valve implantation in patients undergoing hemodialysis
Source: Surg Today. 2023 Sep 5;54(5):428–35. doi: 10.1007/s00595-023-02743-4 (PMC11026236; doi:10.1007/s00595-023-02743-4)
Supplement: Supplementary file 3 — Supplementary file3 (PDF 425 KB) [file 595_2023_2743_MOESM3_ESM.pdf]

Online Resource 3 Characteristics of patients suitable and unsuitable for the TF approach

Variable	Suitable for TF approach (n = 56)	Unsuitable for TF approach (n = 16)	*P*-value
Age, years (SD)	73.0 (6.5)	69.5 (9.4)	.10
Male, n (%)	34 (60.7)	9 (56.3)	.75
BSA, m^2 (SD)	1.46 (0.2)	1.41 (0.2)	.39
SIFAR > 0.95, n (%)	14 (25.0)		
Diabetic nephropathy, n (%)	22 (39.3)	9 (56.3)	.23
HD duration, years [IQR]	9.0 [4.0–16.0]	8.5 [6.3–13.0]	.80
LEAD, n (%)	7 (12.5)	6 (37.5)	.03
Serum calcium, mg/dl (SD)	9.2 (0.8)	9.4 (0.8)	.48
Serum phosphorus, mg/dl (SD)	4.4 (1.7)	4.6 (1.9)	.64

TF, transfemoral; BSA, body surface area; SIFAR, sheath iliofemoral artery ratio; SD, standard deviation;

HD, hemodialysis; LEAD, lower extremity artery disease; IQR, interquartile range
